# Plant Antiviral Defense Disables Other Defenders

**DOI:** 10.1371/journal.pbio.1002327

**Published:** 2015-12-22

**Authors:** Richard Robinson

**Affiliations:** Freelance Science Writer, Sherborn, Massachusetts, United States of America

## Abstract

A new study shows that when plants are infected by viruses they express an RNase enzyme that digests the double-stranded precursors of small antiviral RNAs. Read the accompanying Research Article.

One of the most extraordinary developments in molecular biology in the last quarter century has been the growing understanding of the role of small RNA molecules. Originally described as oddities of petunia plants, these noncoding RNAs, mostly 21 to 24 nucleotides long, are now recognized to be ubiquitous in the cells of both plants and animals, as well as many fungi, where they trigger destruction of target RNAs, regulating gene expression and defending against viral infections.

In plants, small RNAs are produced from long double-stranded RNAs (dsRNAs) by the actions of the DICER-LIKE enzymes, members of the RNAseIII family. Plants also produce another group of RNAseIII enzymes, called RNASE THREE-LIKE (RTL), but their function is less clear. In a new study in *PLOS Biology*, Nahid Shamandi, Matthias Zytnicki, Hervé Vaucheret, and colleagues show that in response to viral infection, one member of the group, RTL1, represses production of small RNAs. While this action may once have been a valuable antiviral response, it now appears to be counterproductive, disabling other antiviral systems before becoming disabled itself by viral proteins.

Studies in the model plant *Arabidopsis thaliana* have indicated that RTL1 is expressed weakly in plant roots, and elsewhere barely at all. But its RNAseIII activity suggested to the authors it may play a role in viral defense, a supposition borne out by the finding that levels of RTL1 protein rose twenty-fold after plants were infected with any one of several common plant viruses. In otherwise healthy plants, overexpression of RTL1 suppressed production of small RNAs from over 6,000 loci, representing the vast majority of those examined, including multiple classes of small interfering RNAs (siRNAs), known for their roles in fighting viral infections.

Overexpression of RTL1 reduced the various siRNA species by an even greater degree than did deleting the DICER-LIKE enzymes, suggesting that RTL1 did not exert its effect by inhibiting those enzymes. Rather, the authors hypothesized that RTL1 might cleave the dsRNA precursors of the various siRNAs, preventing them from being processed by the DCLs at all. While mutating the DICER-LIKE enzymes in wild-type plants led one such precursor to accumulate as expected, overexpression of RTL1 prevented that accumulation, indicating it was indeed degrading it upstream of the DICER-LIKE enzymes.

Long dsRNAs are produced by viruses during their replication, and so their cleavage by RTL1 might lead to an overall improvement in survival for a cell under attack if RTL1 has access to these viral dsRNAs. However, viral long dsRNAs are also processed by the DICER-LIKE enzymes, and the resulting siRNAs guide the cleavage of viral RNAs into fragments that are transformed into dsRNAs by cellular enzymes to amplify the plant defenses. RTL1 can also cleave these long dsRNAs, thus disabling this antiviral defense. Unfortunately for Arabidopsis, most viruses contain their own anti-antiviral weapons, called viral suppressors of RNA silencing (VSRs), among them a protein called 2b, which is known to inhibit a key enzyme in the siRNA pathway called AGO1. Here, the authors found that 2b, along with several other known VSRs, also inhibited RTL1, and plants overexpressing RTL1 fared no better than wild-type plants in fending off viral infection. Moreover, viruses that do not express a VSR capable of inhibiting RTL1 appear to escape degradation by RTL1 and instead use RTL1 to knock-down the plant antiviral defense (Fig 1).

**Fig 1:**
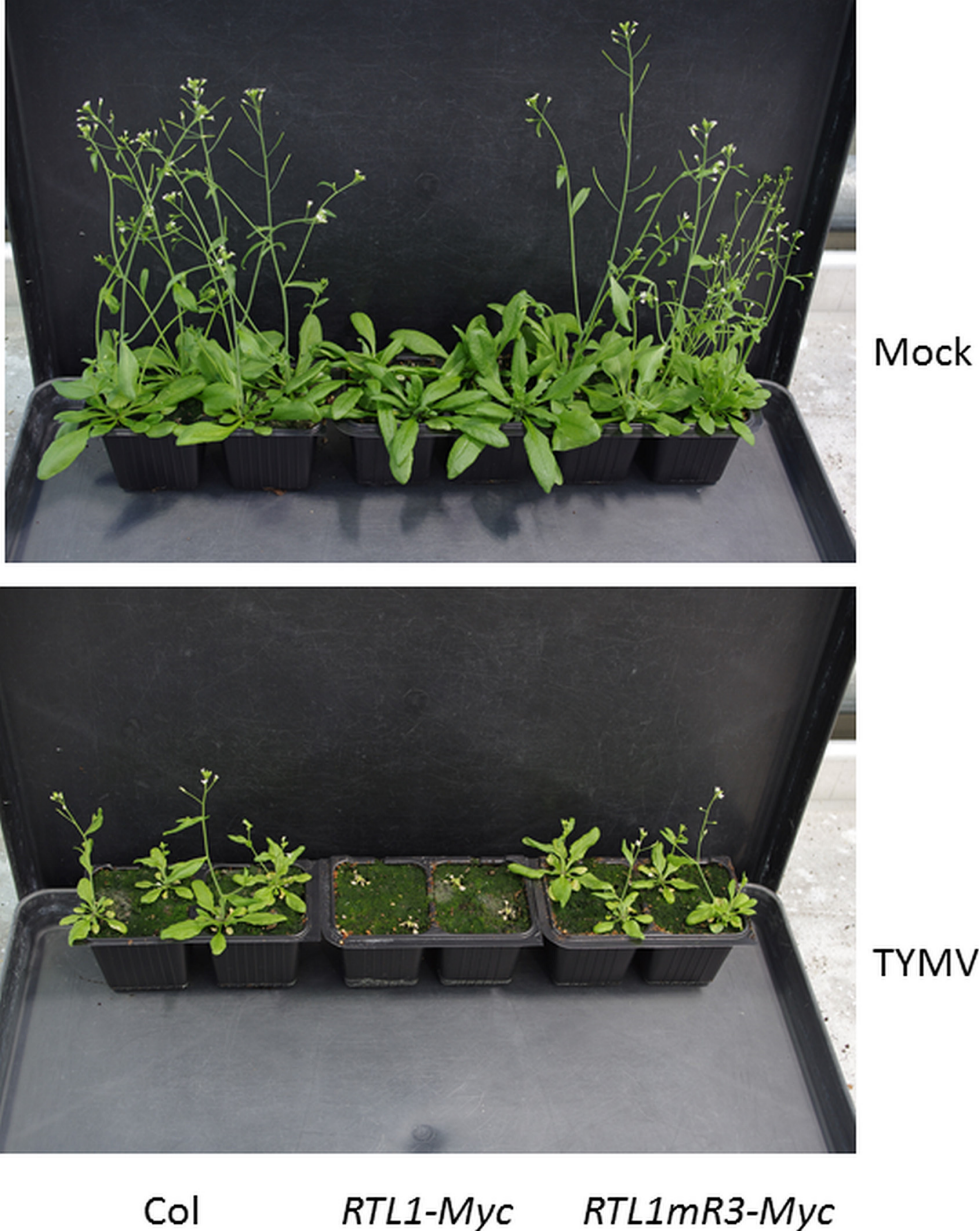
Hypersusceptibility of plants overexpressing RTL1 to viruses that do not express a VSR capable of inhibiting RTL1. Compared to wild-type plants (Col) or plants overexpressing a nonfunctional RTL1 (RTL1mR3-Myc), plants overexpressing a functional RTL1 (RTL1-Myc) develop normally despite a late flowering phenotype (top image). However, they are hypersusceptible to infection by turnip yellow mosaic virus (TYMV), a virus that that does not express a VSR capable of inhibiting RTL1 activity (bottom image). *Image credit*: *Nahid Shamandi*.

So what good is RTL1? The question remains open. It is possible, though speculative, that we are looking at a snapshot in the coevolution of viruses and plants, in which RTL1 evolved to serve as a second line of defense but has been outmatched by more recently evolved viral countermeasures. But the authors point out that the gene is conserved in plants, and no naturally occurring mutants are known, suggesting it likely has important functions remaining to be discovered.

## Abbreviations

dsRNA: double-stranded RNA
RTL: RNASE THREE-LIKE
siRNA: small interfering RNA
VSR: viral suppressor or RNA silencing
